# Supplementary figures and images for: Androglobin, a chimeric mammalian globin, is required for male fertility
Source: eLife. 2022 Jun 14;11:e72374. doi: 10.7554/eLife.72374 (PMC9249397; doi:10.7554/eLife.72374)

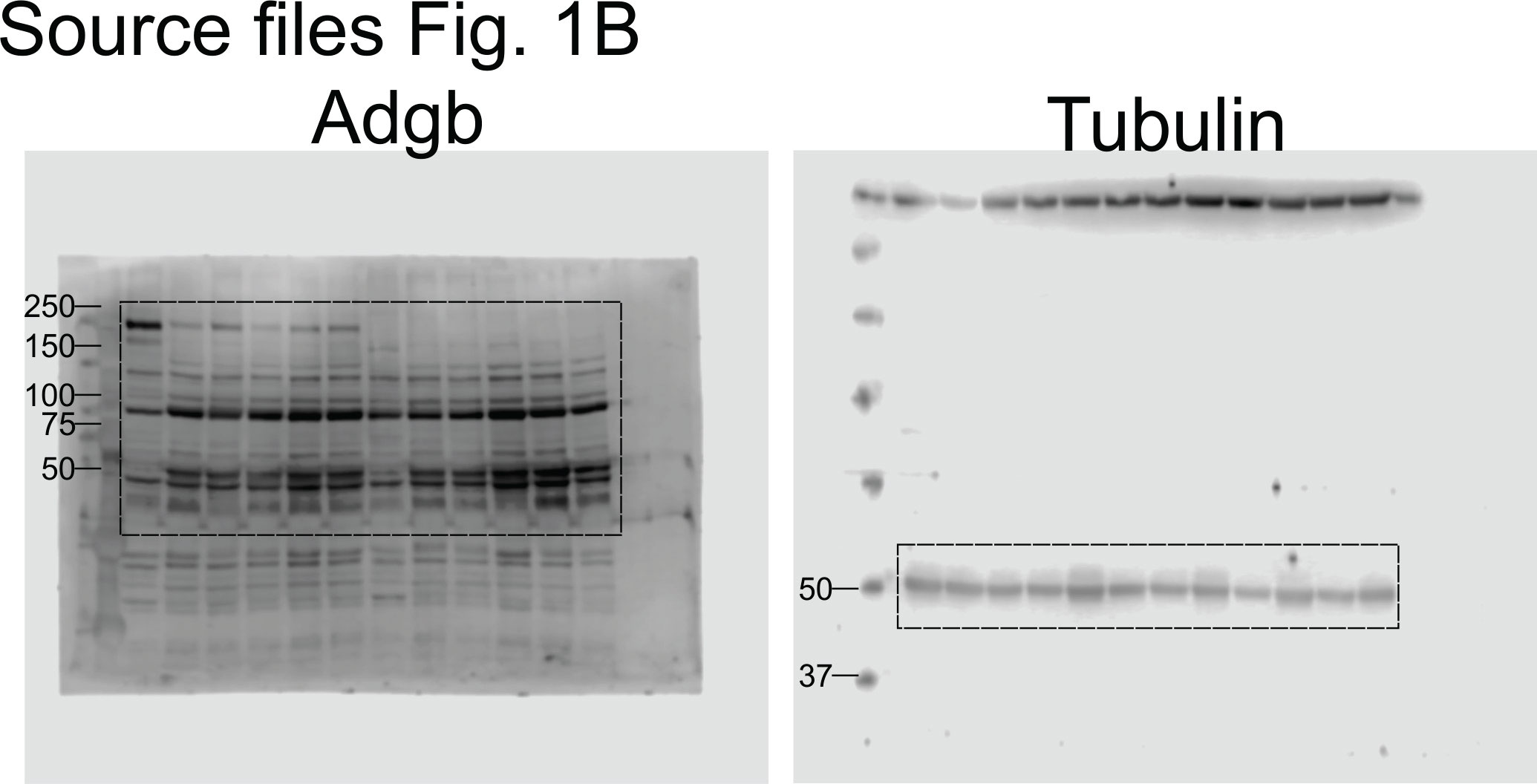

Supplement: Figure 1—source data 1. [file elife-72374-fig1-data1.zip › Figure 1-source data 1.jpg]

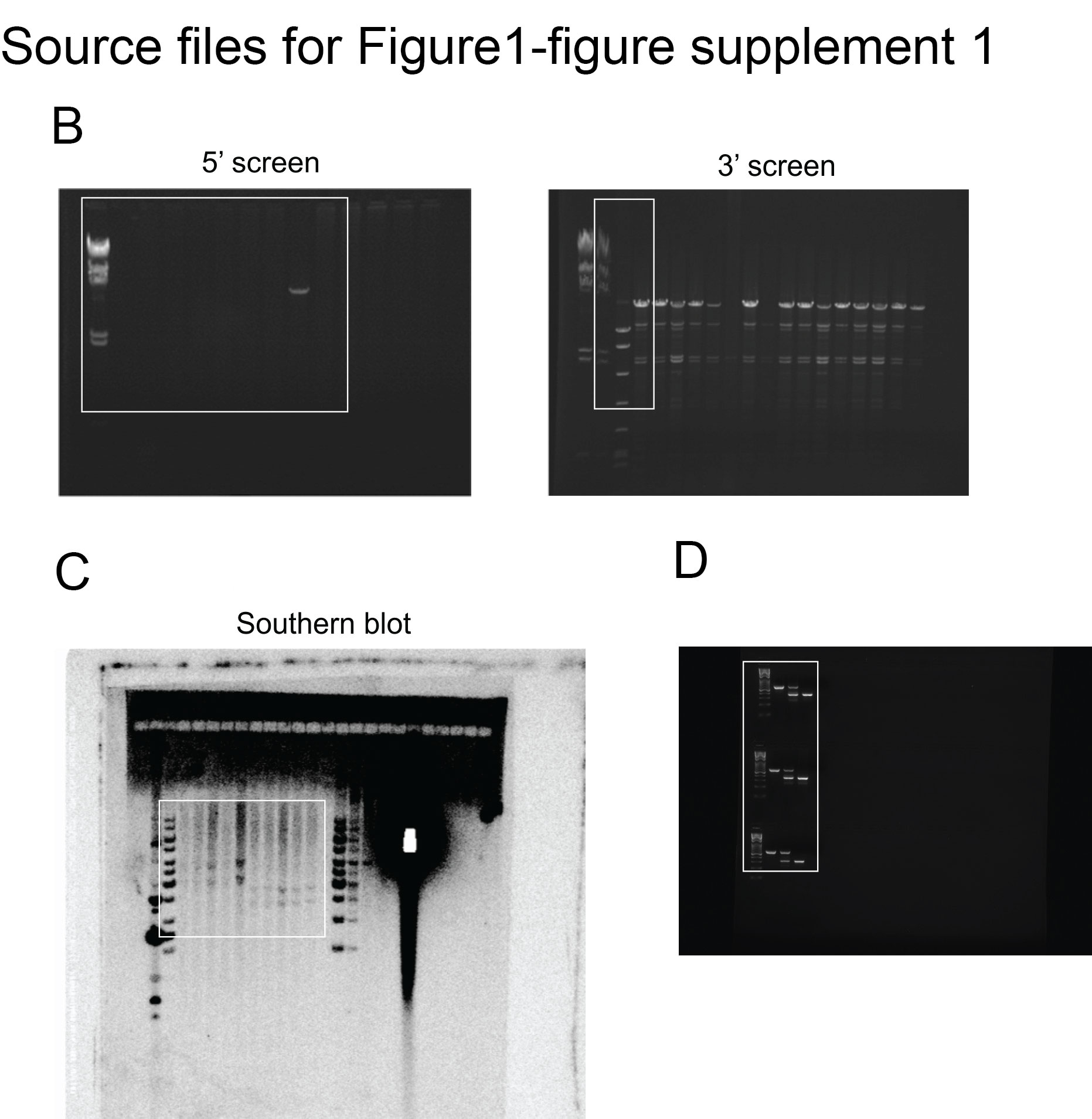

Supplement: Figure 1—figure supplement 1—source data 1. [file elife-72374-fig1-figsupp1-data1.zip › Figure 1-figure supplement 1-source data 1.jpg]

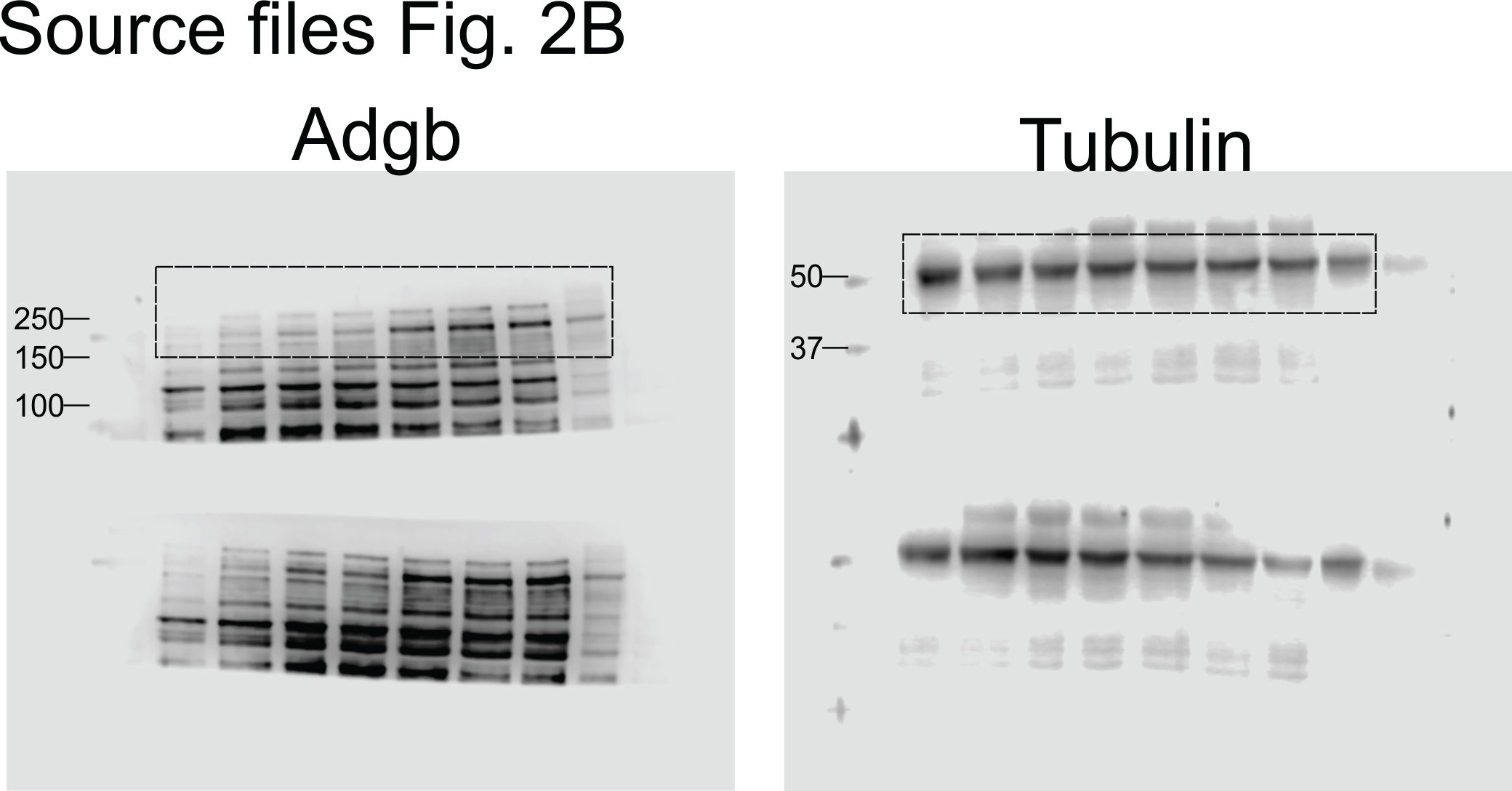

Supplement: Figure 2—source data 1. [file elife-72374-fig2-data1.zip › Figure 2-source data 1.jpg]

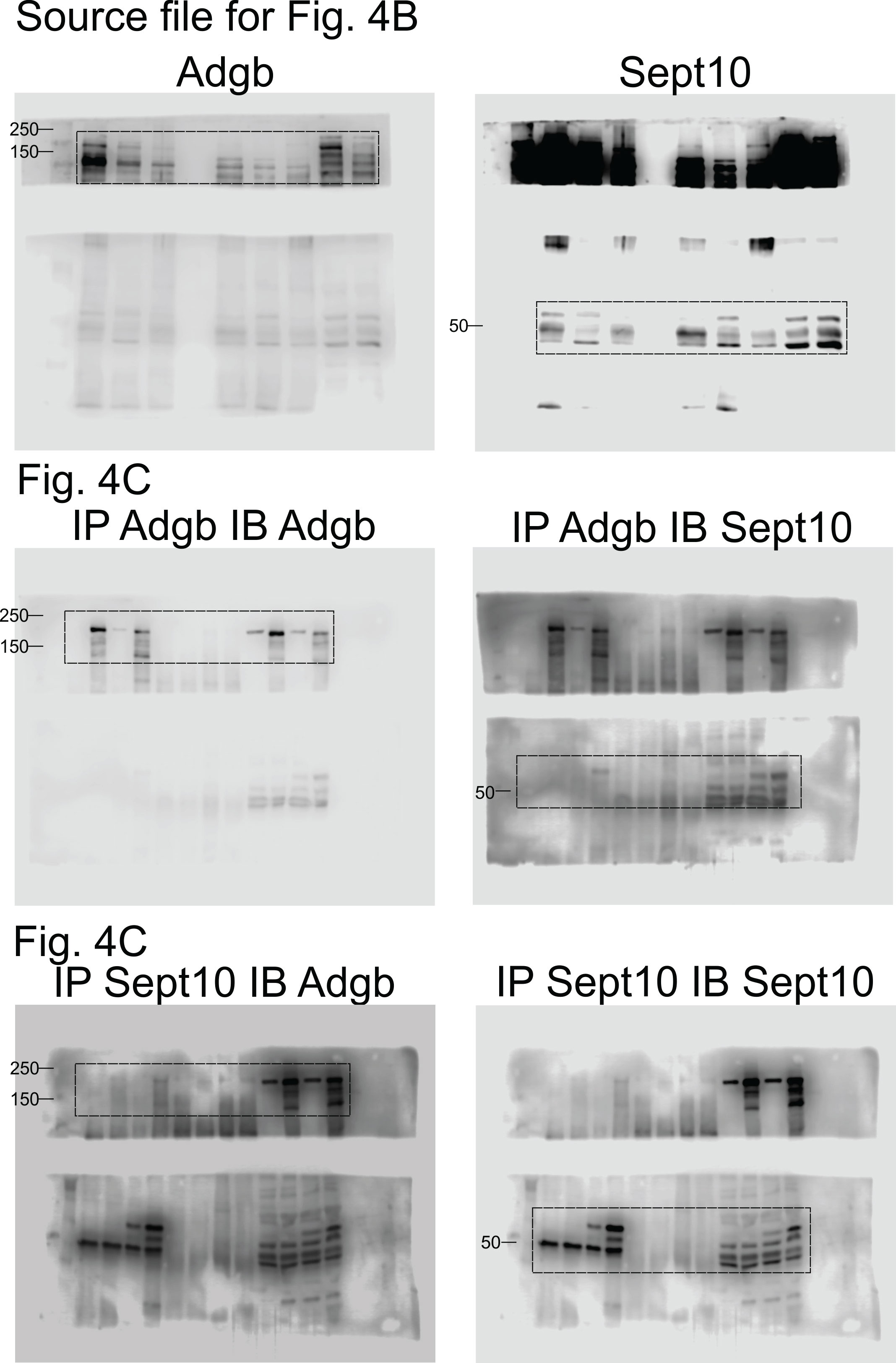

Supplement: Figure 4—source data 1. [file elife-72374-fig4-data1.zip › Figure 4-source data 1B.jpg]

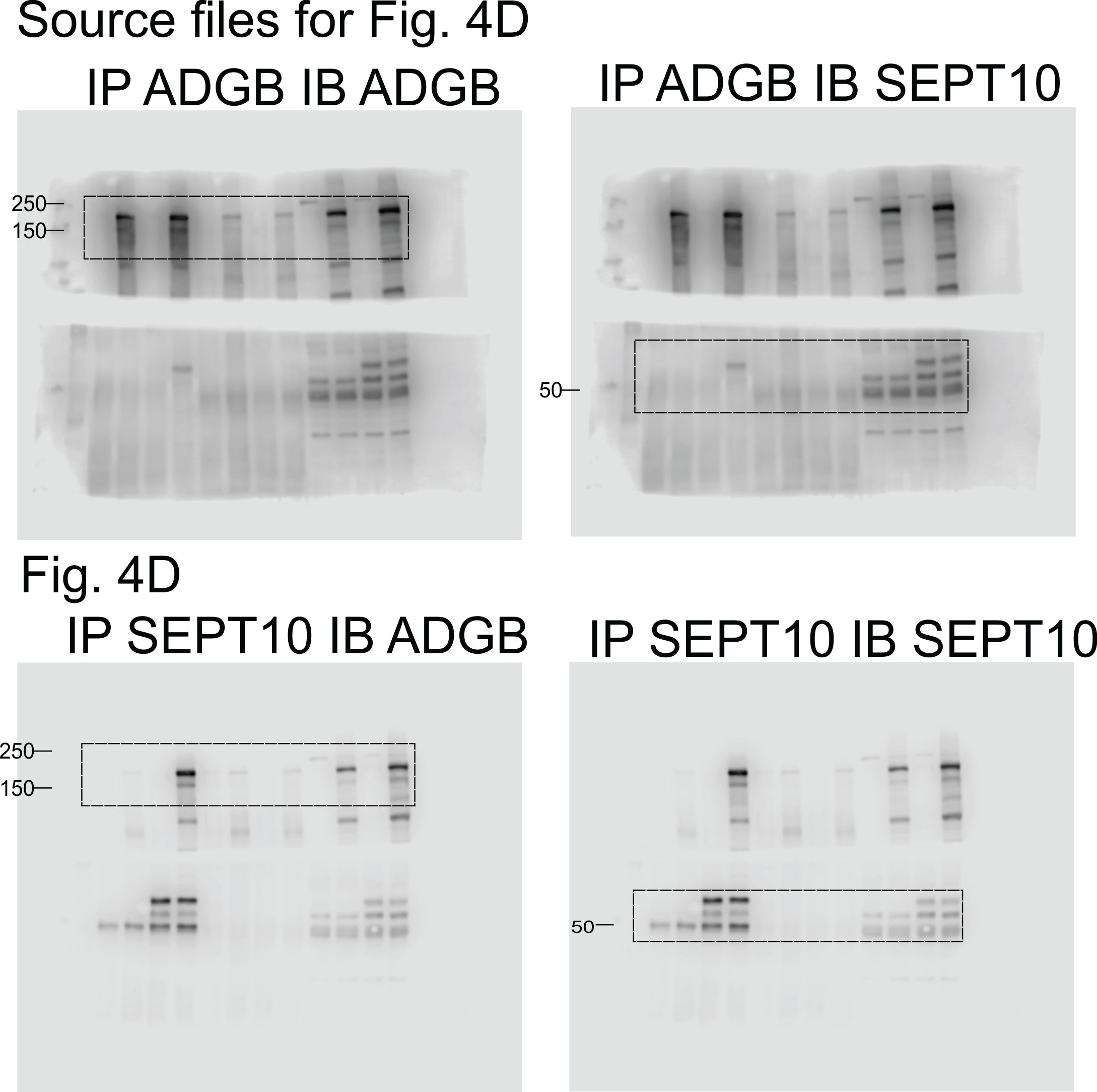

Supplement: Figure 4—source data 2. [file elife-72374-fig4-data2.zip › Figure 4-source data 1D.jpg]

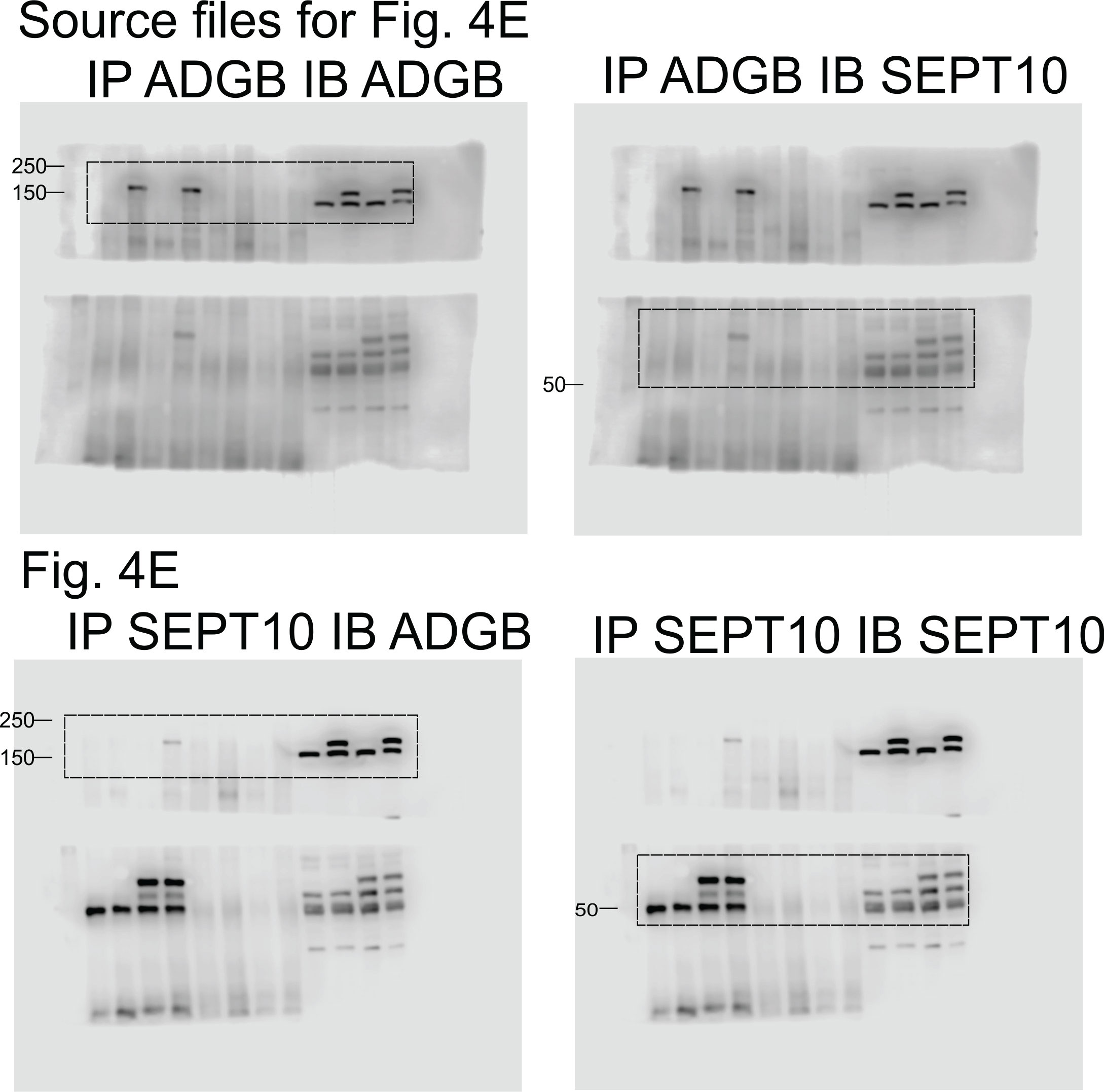

Supplement: Figure 4—source data 3. [file elife-72374-fig4-data3.zip › Figure 4-source data 1E.jpg]

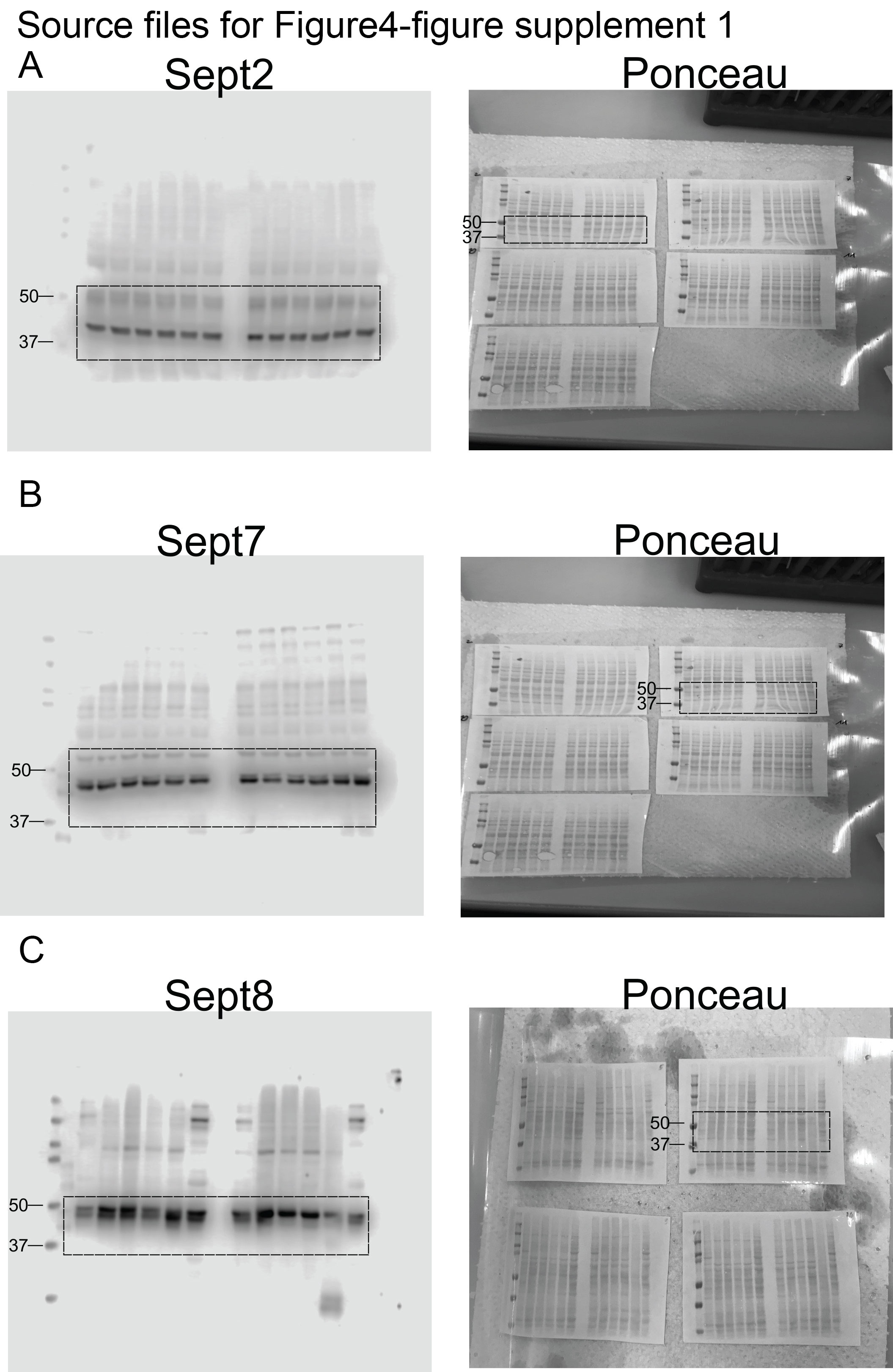

Supplement: Figure 4—figure supplement 1—source data 1. [file elife-72374-fig4-figsupp1-data1.zip › Figure 4—figure supplement 1—source data 1.jpg]

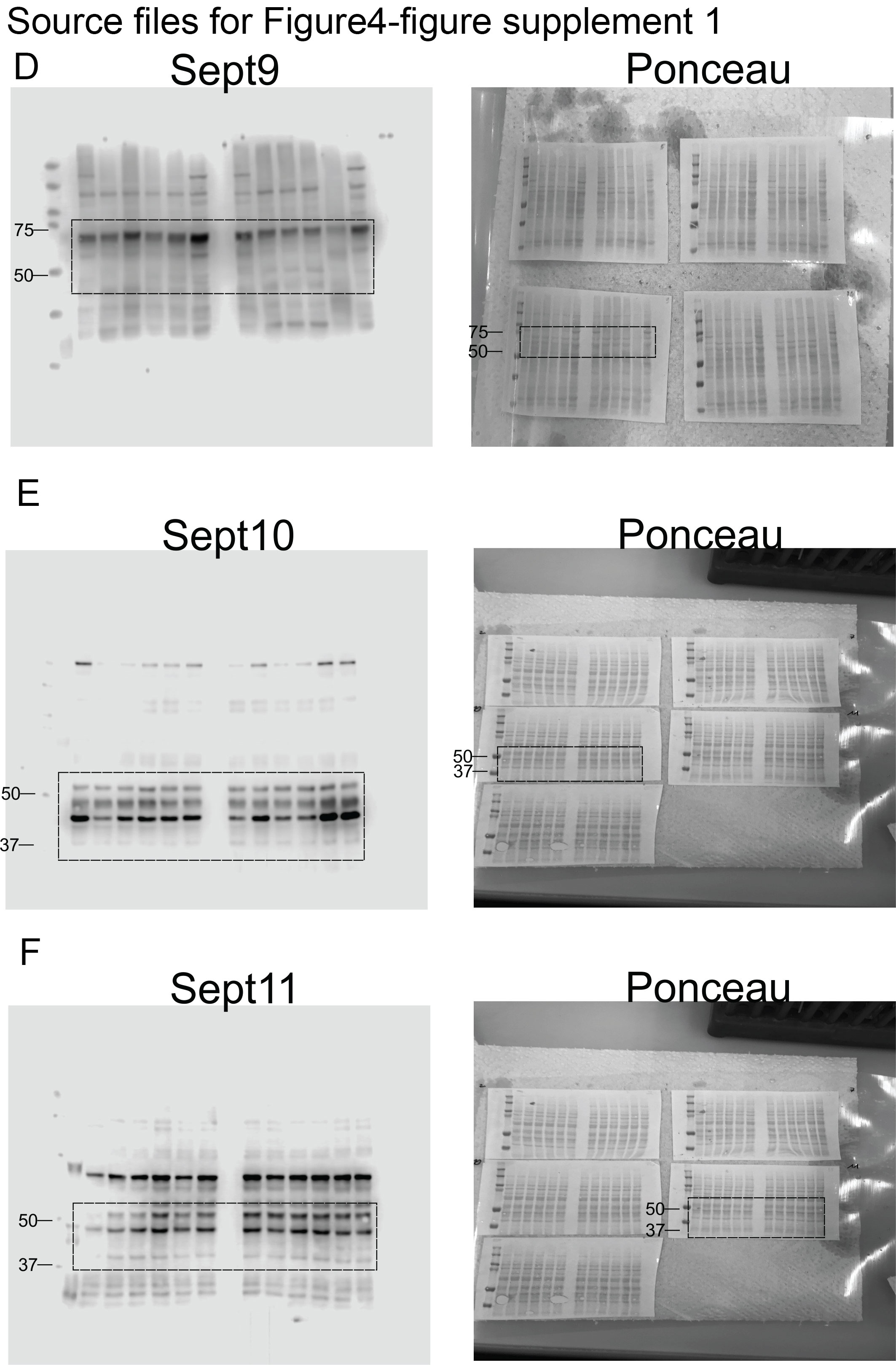

Supplement: Figure 4—figure supplement 1—source data 2. [file elife-72374-fig4-figsupp1-data2.zip › Figure 4—figure supplement 1—source data 2.jpg]

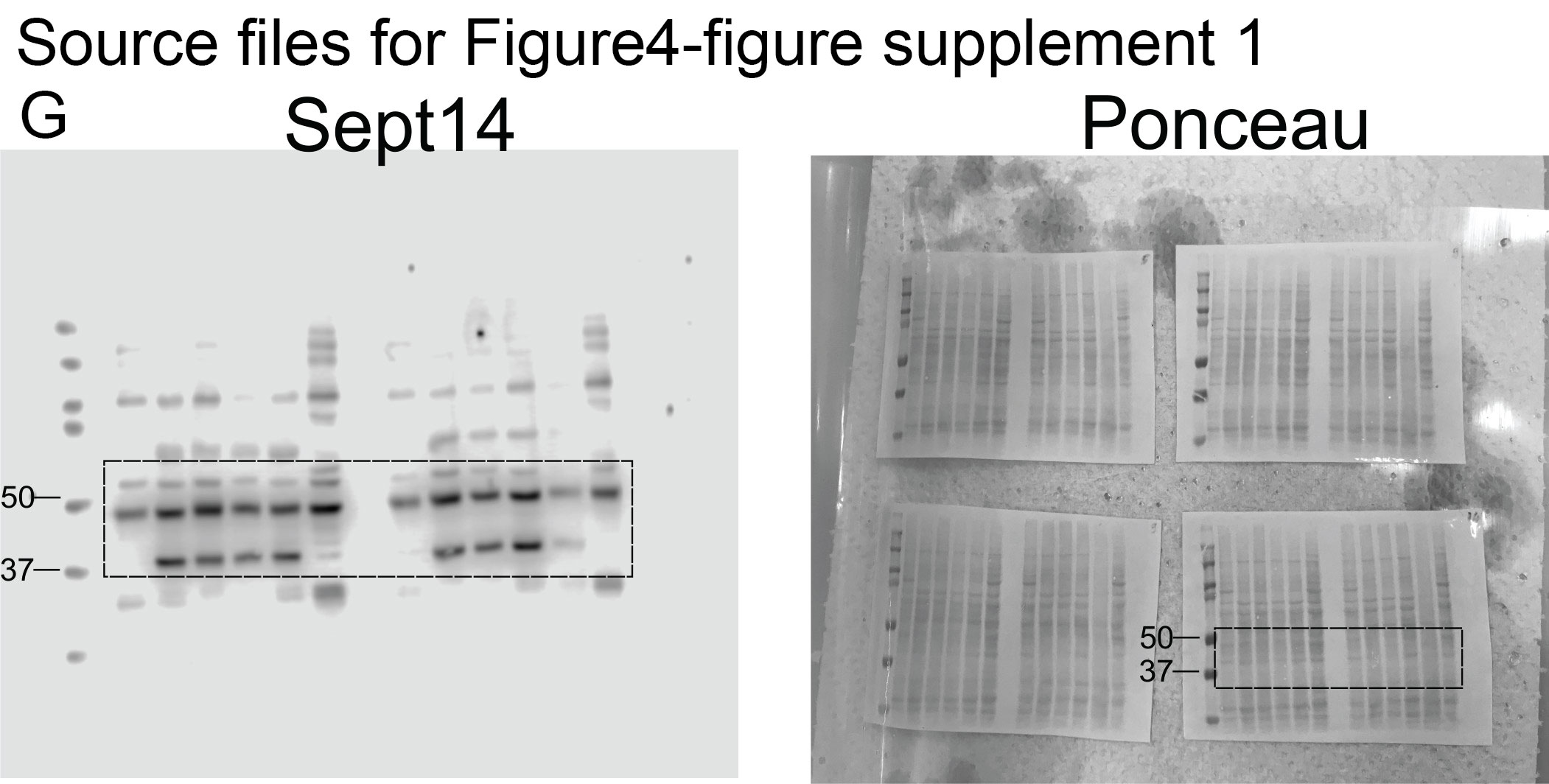

Supplement: Figure 4—figure supplement 1—source data 3. [file elife-72374-fig4-figsupp1-data3.zip › Figure 4—figure supplement 1—source data 3.jpg]

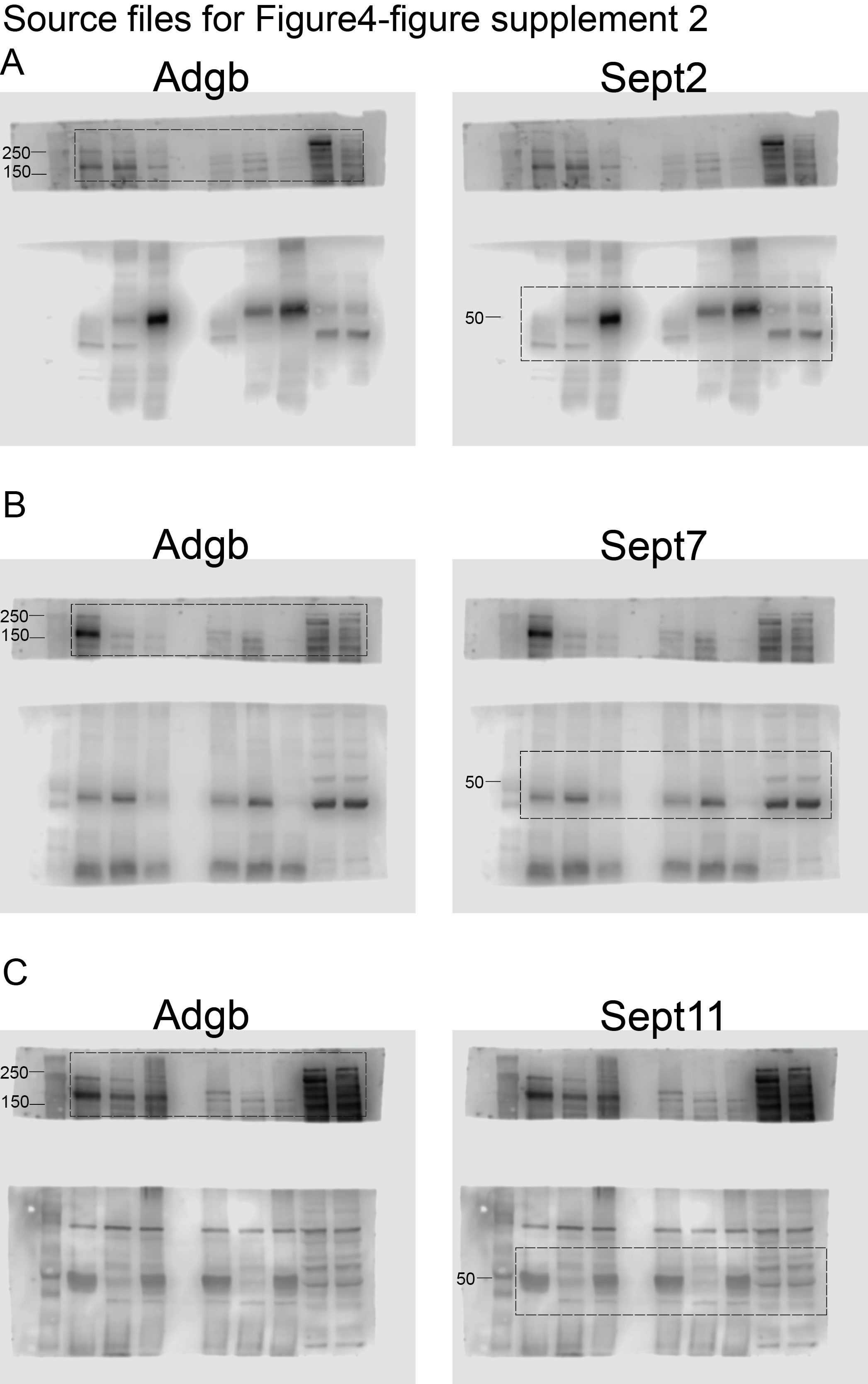

Supplement: Figure 4—figure supplement 2—source data 1. [file elife-72374-fig4-figsupp2-data1.zip › Figure 4—figure supplement 2—source data 1.jpg]

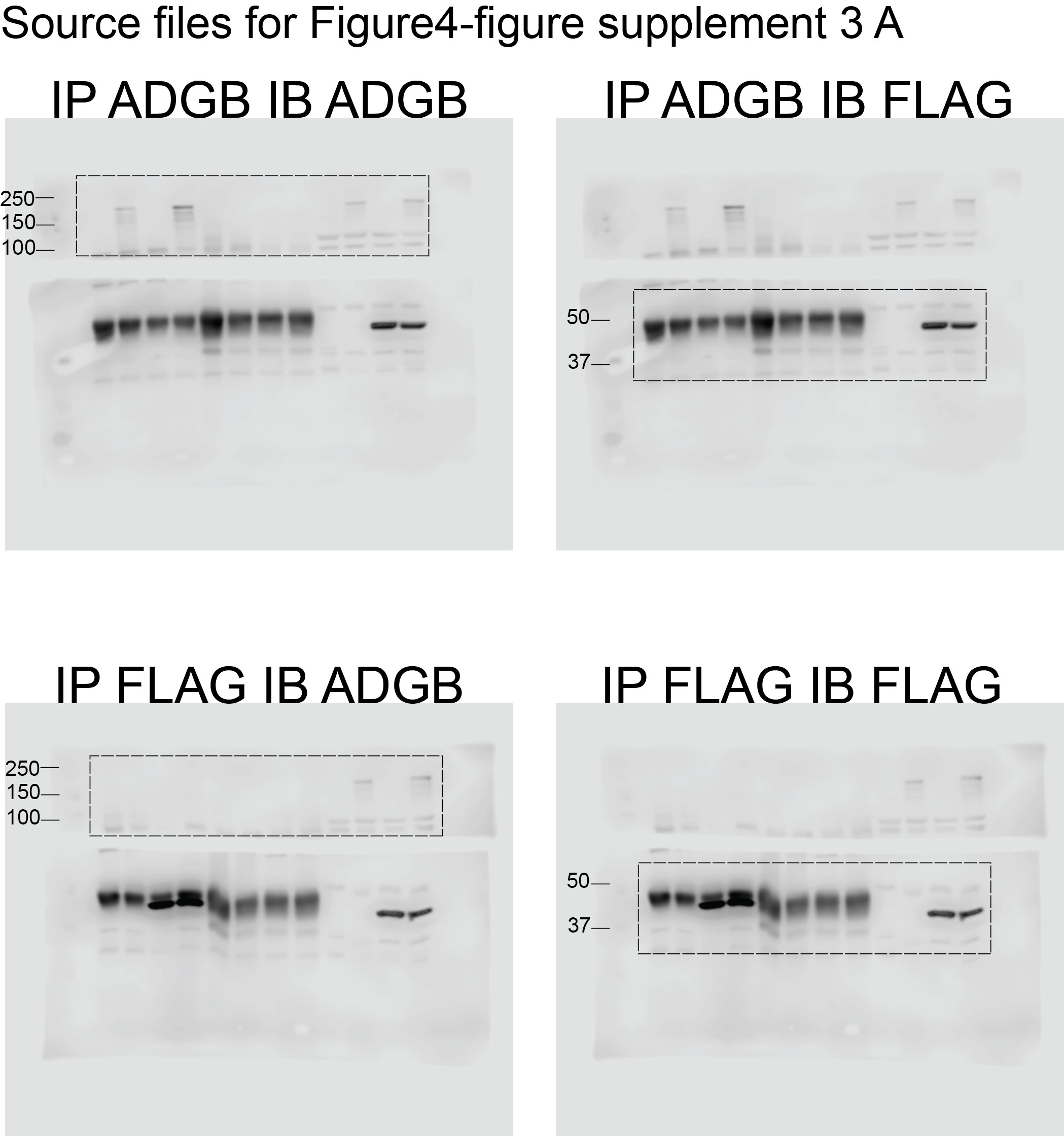

Supplement: Figure 4—figure supplement 3—source data 1. [file elife-72374-fig4-figsupp3-data1.zip › Figure 4—figure supplement 3—source data 1.jpg]

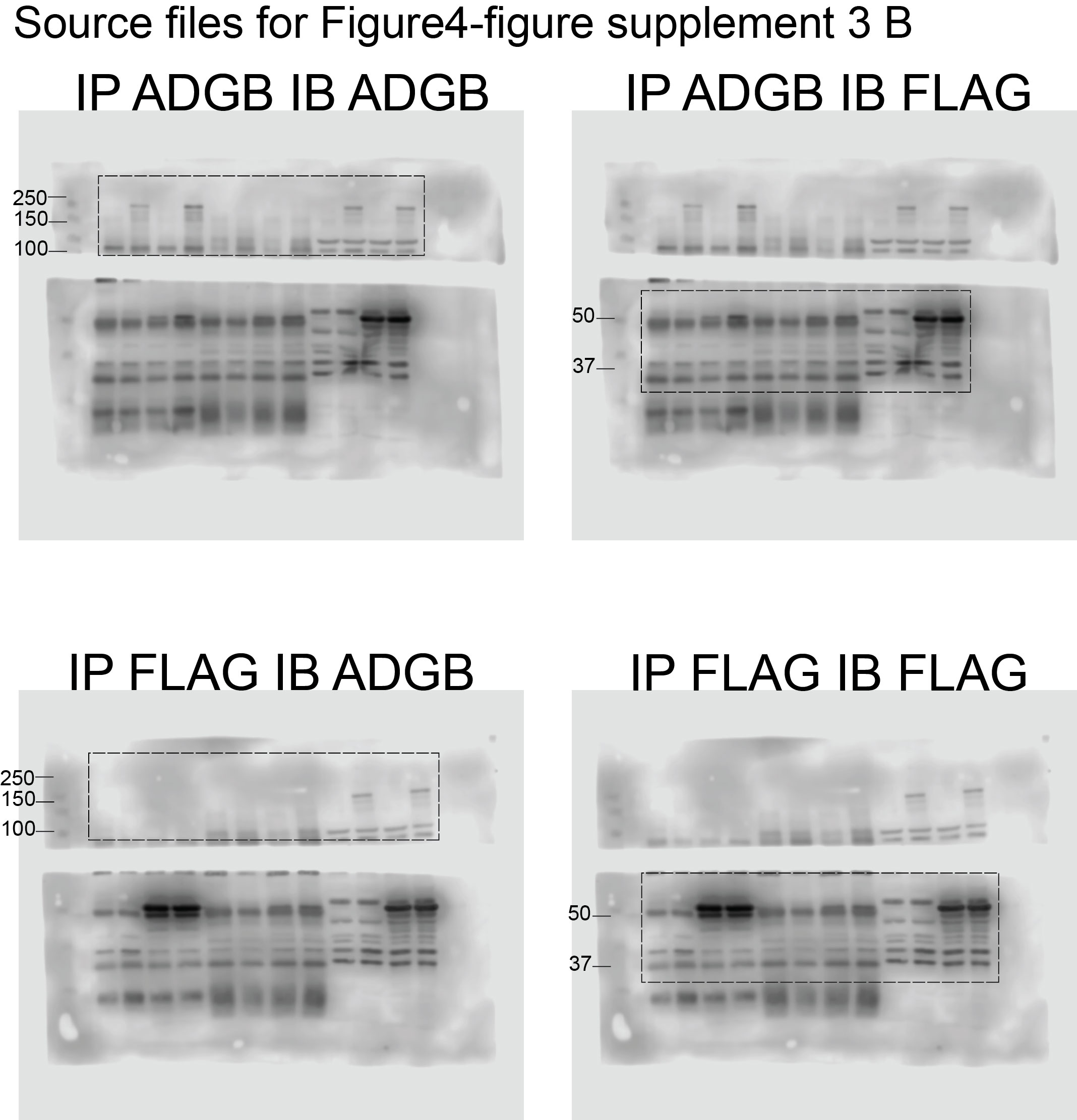

Supplement: Figure 4—figure supplement 3—source data 2. [file elife-72374-fig4-figsupp3-data2.zip › Figure 4—figure supplement 3—source data 2B.jpg]

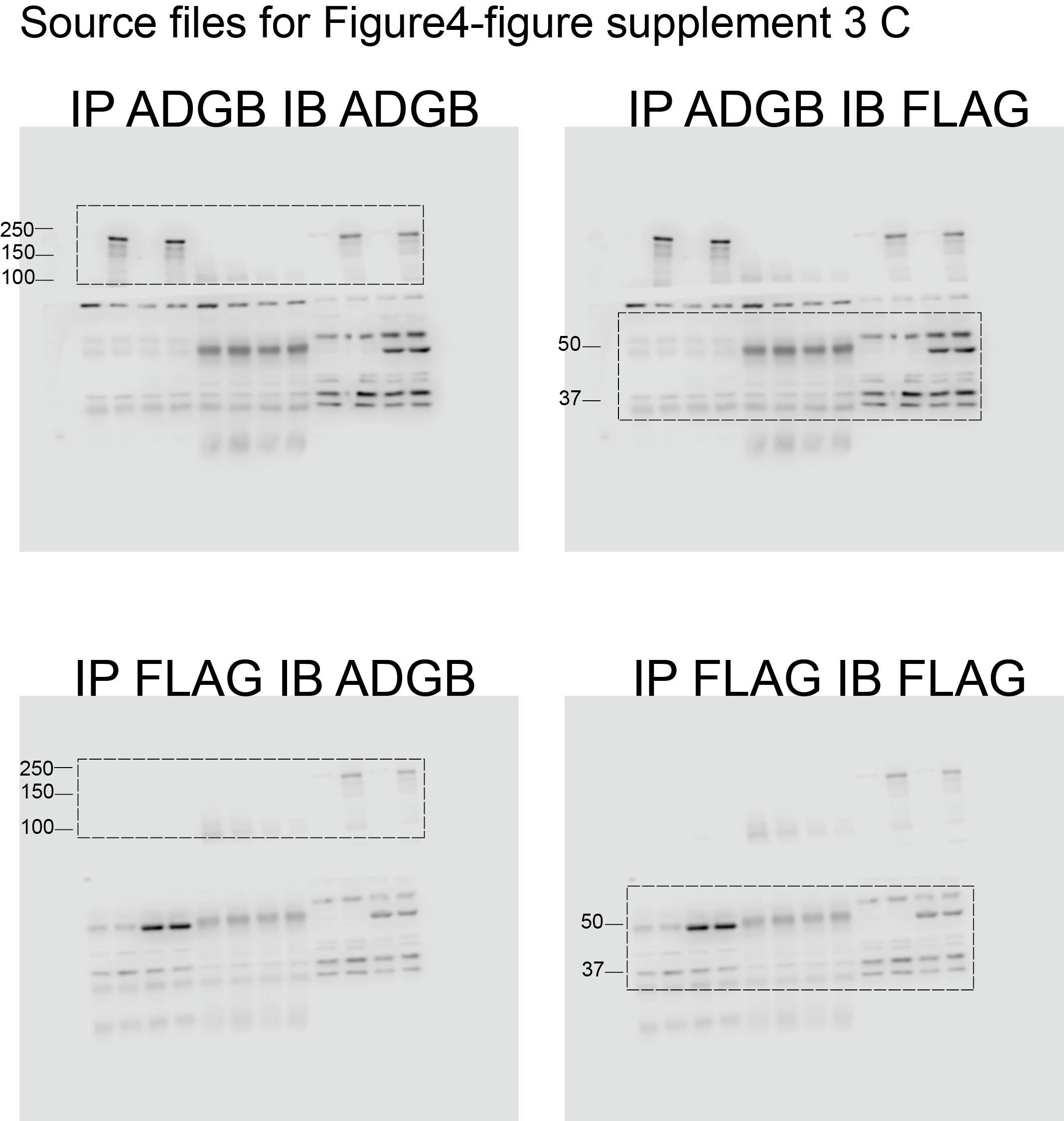

Supplement: Figure 4—figure supplement 3—source data 3. [file elife-72374-fig4-figsupp3-data3.zip › Figure 4—figure supplement 3—source data 3C.jpg]

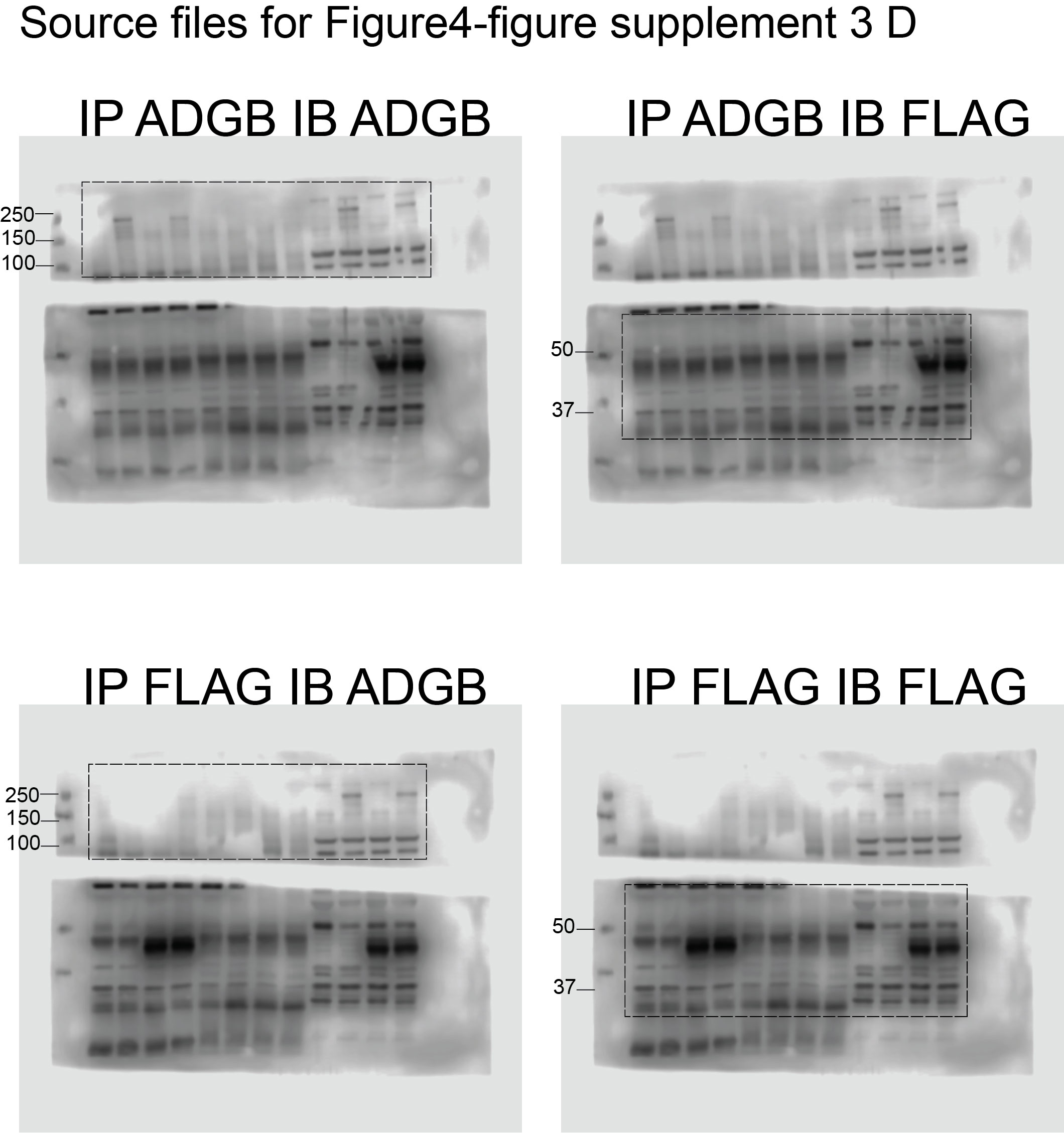

Supplement: Figure 4—figure supplement 3—source data 4. [file elife-72374-fig4-figsupp3-data4.zip › Figure 4—figure supplement 3—source data 4D.jpg]

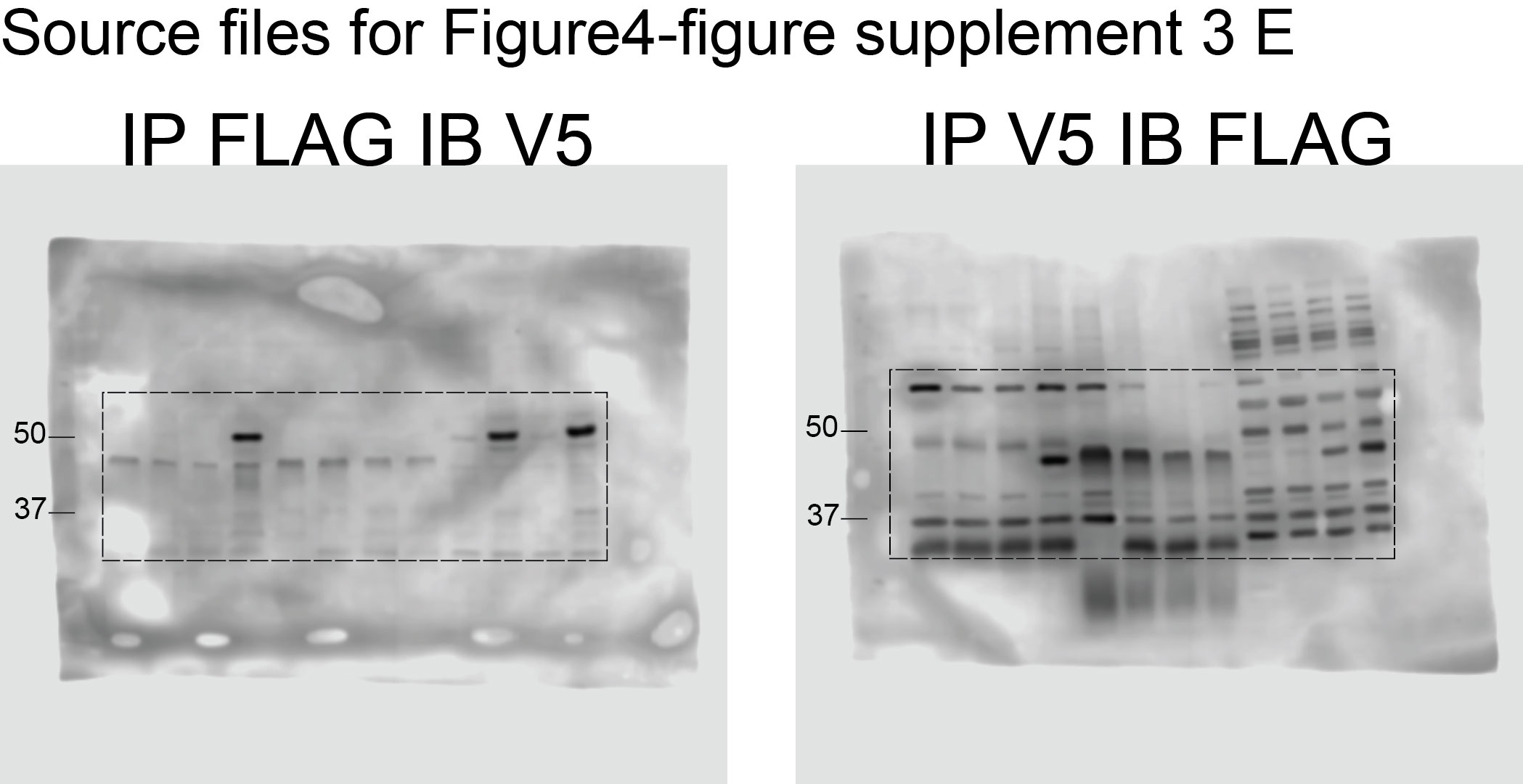

Supplement: Figure 4—figure supplement 3—source data 5. [file elife-72374-fig4-figsupp3-data5.zip › Figure 4—figure supplement 3—source data 5.jpg]

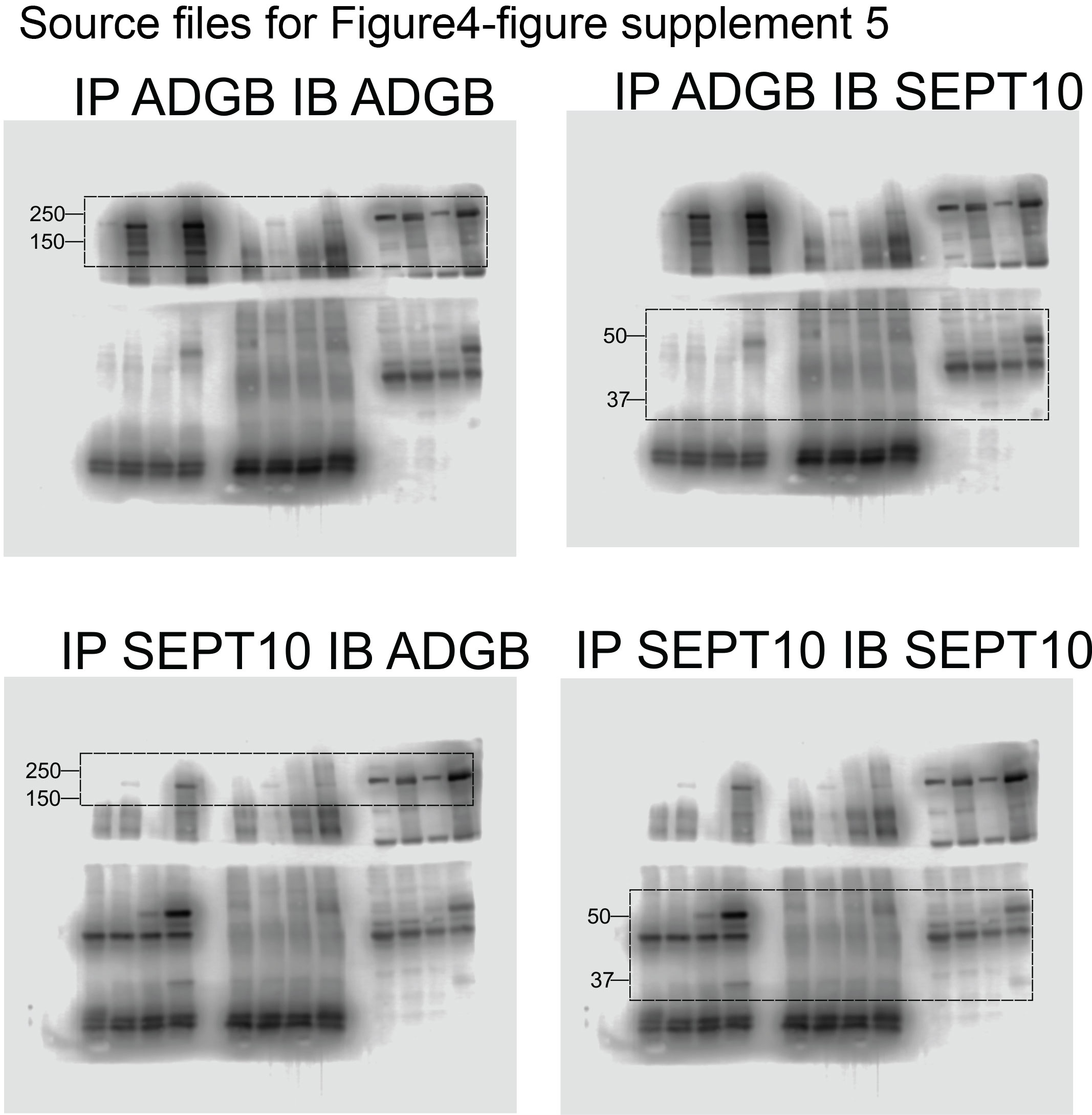

Supplement: Figure 4—figure supplement 5—source data 1. [file elife-72374-fig4-figsupp5-data1.zip › Figure 4—figure supplement 5—source data 1.jpg]

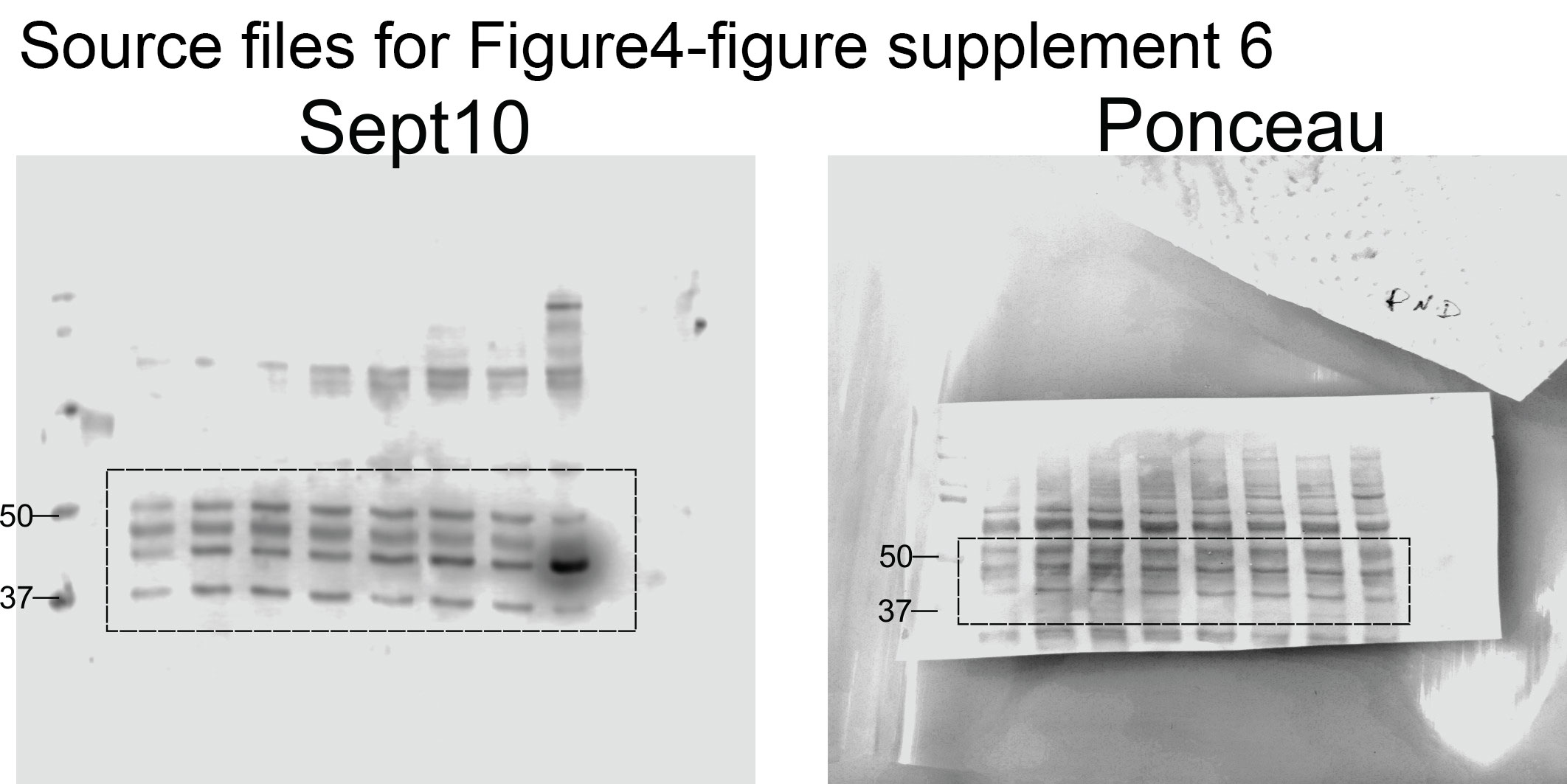

Supplement: Figure 4—figure supplement 6—source data 1. [file elife-72374-fig4-figsupp6-data1.zip › Figure 4—figure supplement 6—source data 1.jpg]

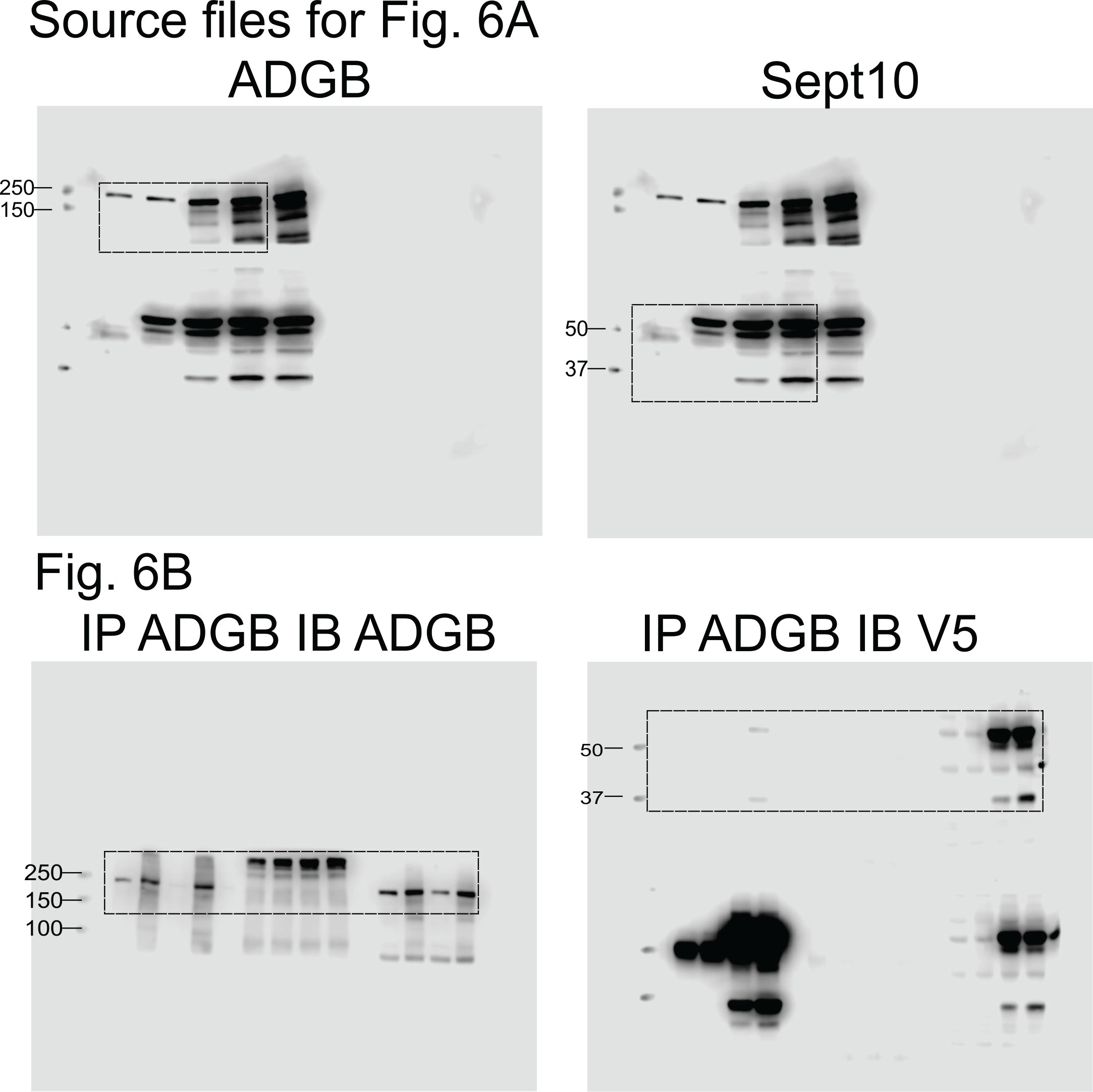

Supplement: Figure 6—source data 1. [file elife-72374-fig6-data1.jpg]

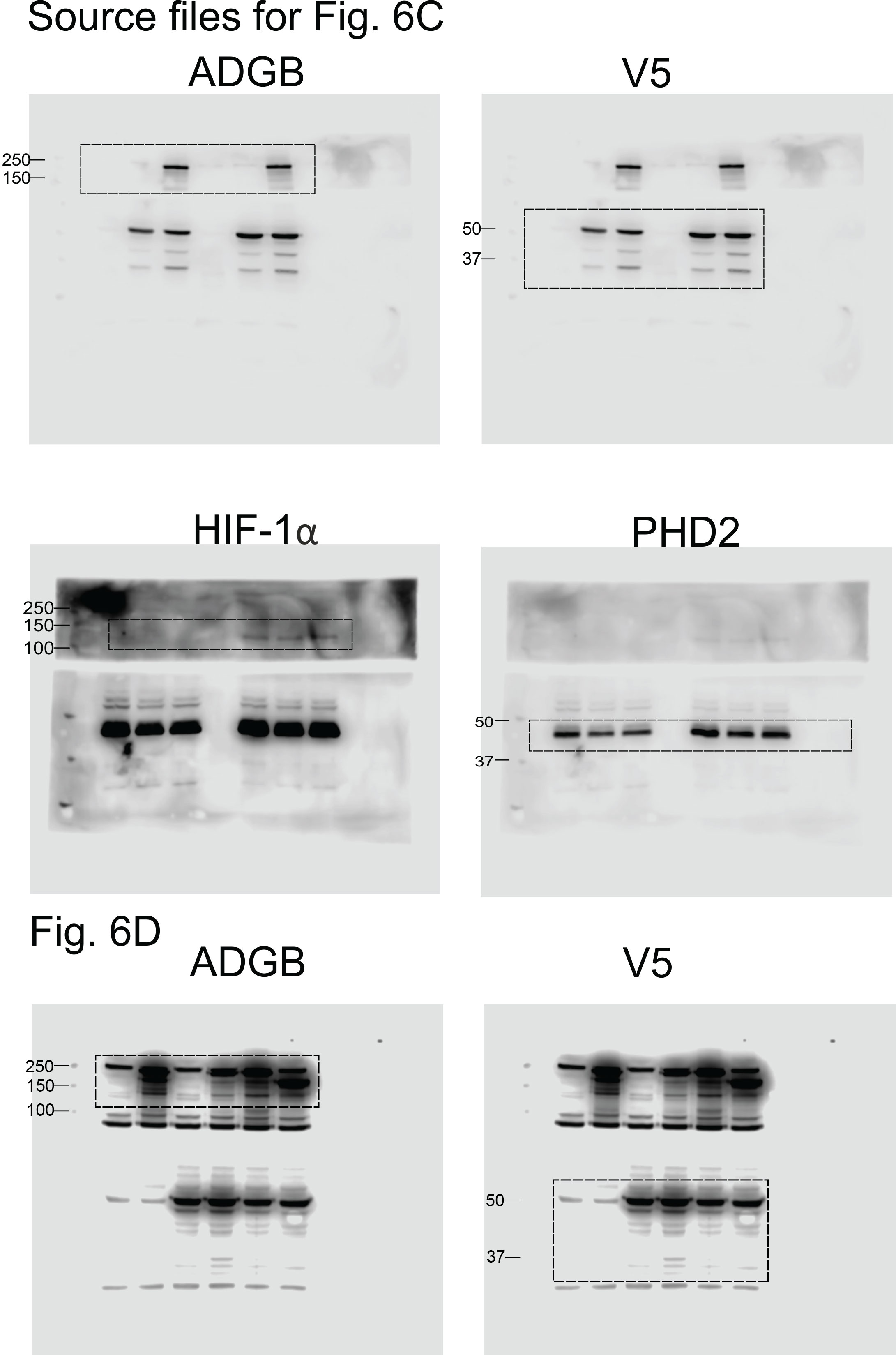

Supplement: Figure 6—source data 2. [file elife-72374-fig6-data2.jpg]

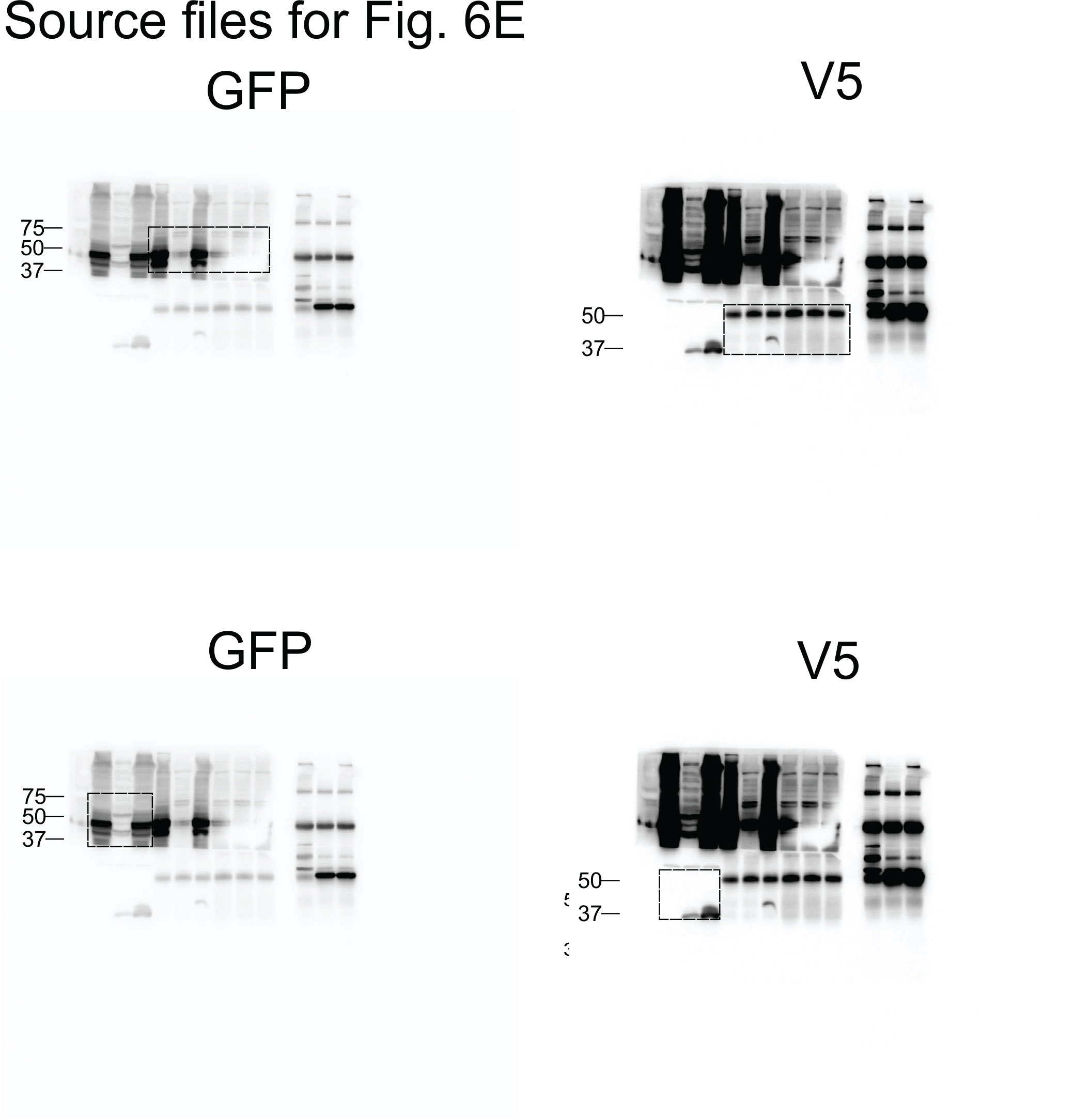

Supplement: Figure 6—source data 3. [file elife-72374-fig6-data3.jpg]

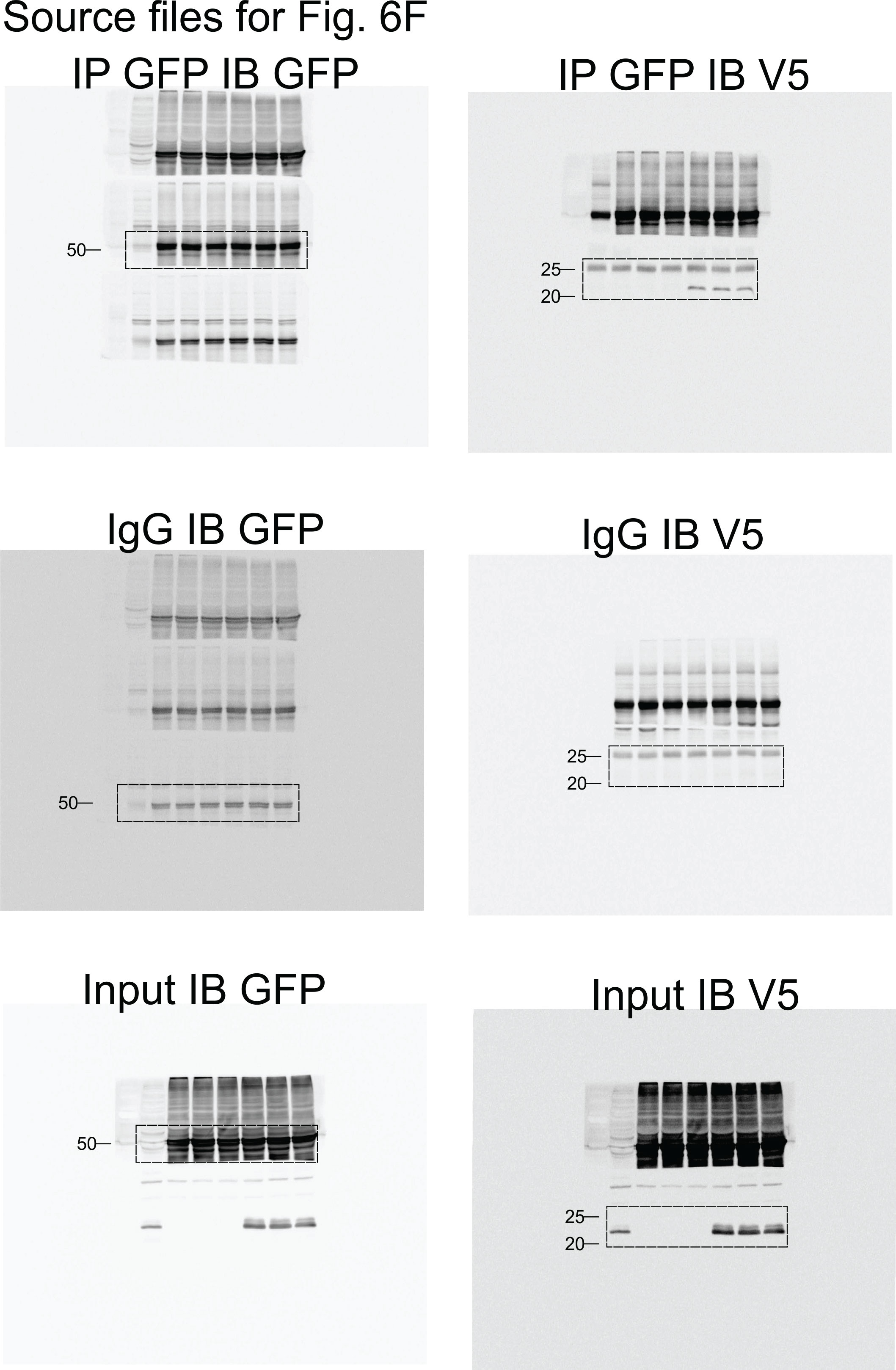

Supplement: Figure 6—source data 4. [file elife-72374-fig6-data4.jpg]

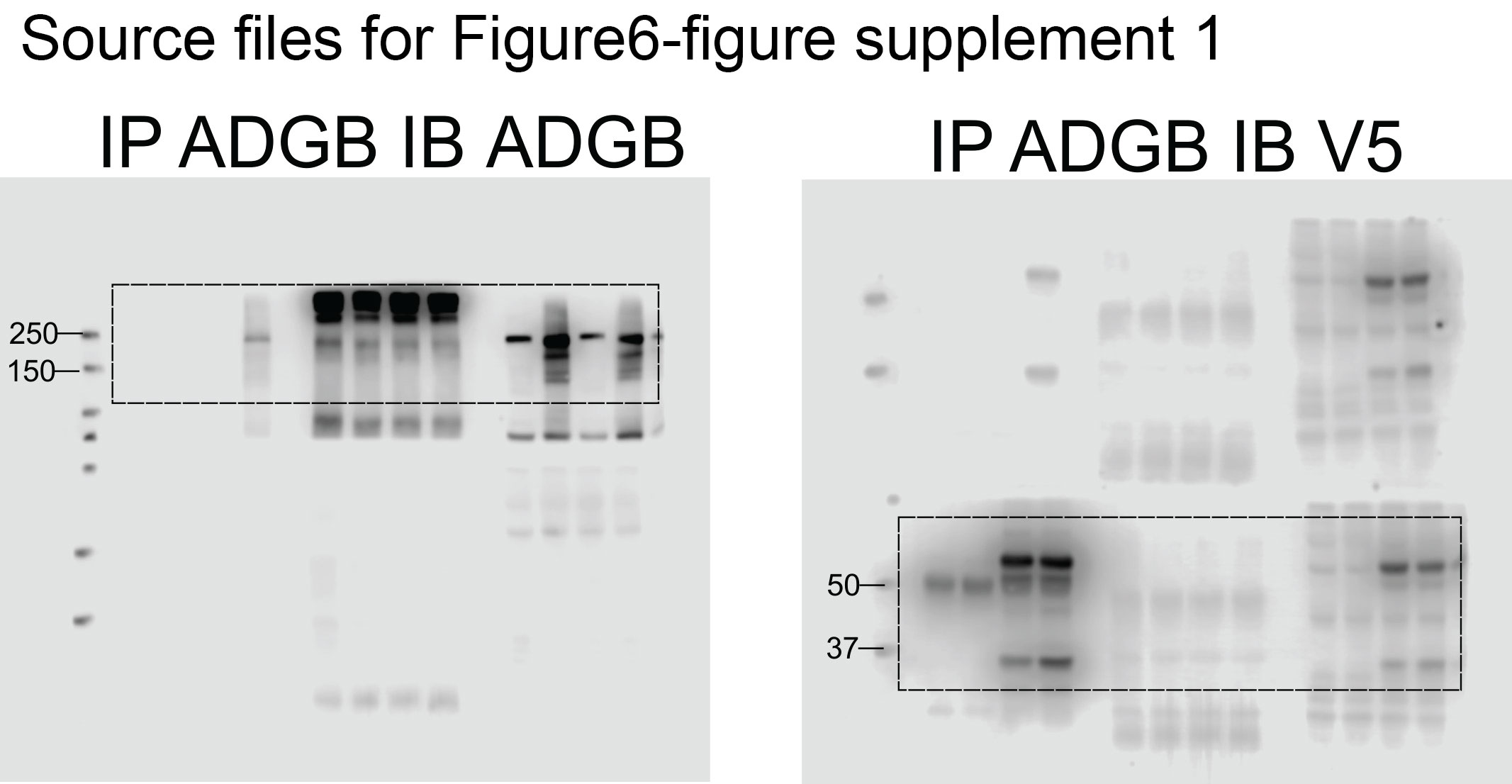

Supplement: Figure 6—figure supplement 1—source data 1. [file elife-72374-fig6-figsupp1-data1.jpg]

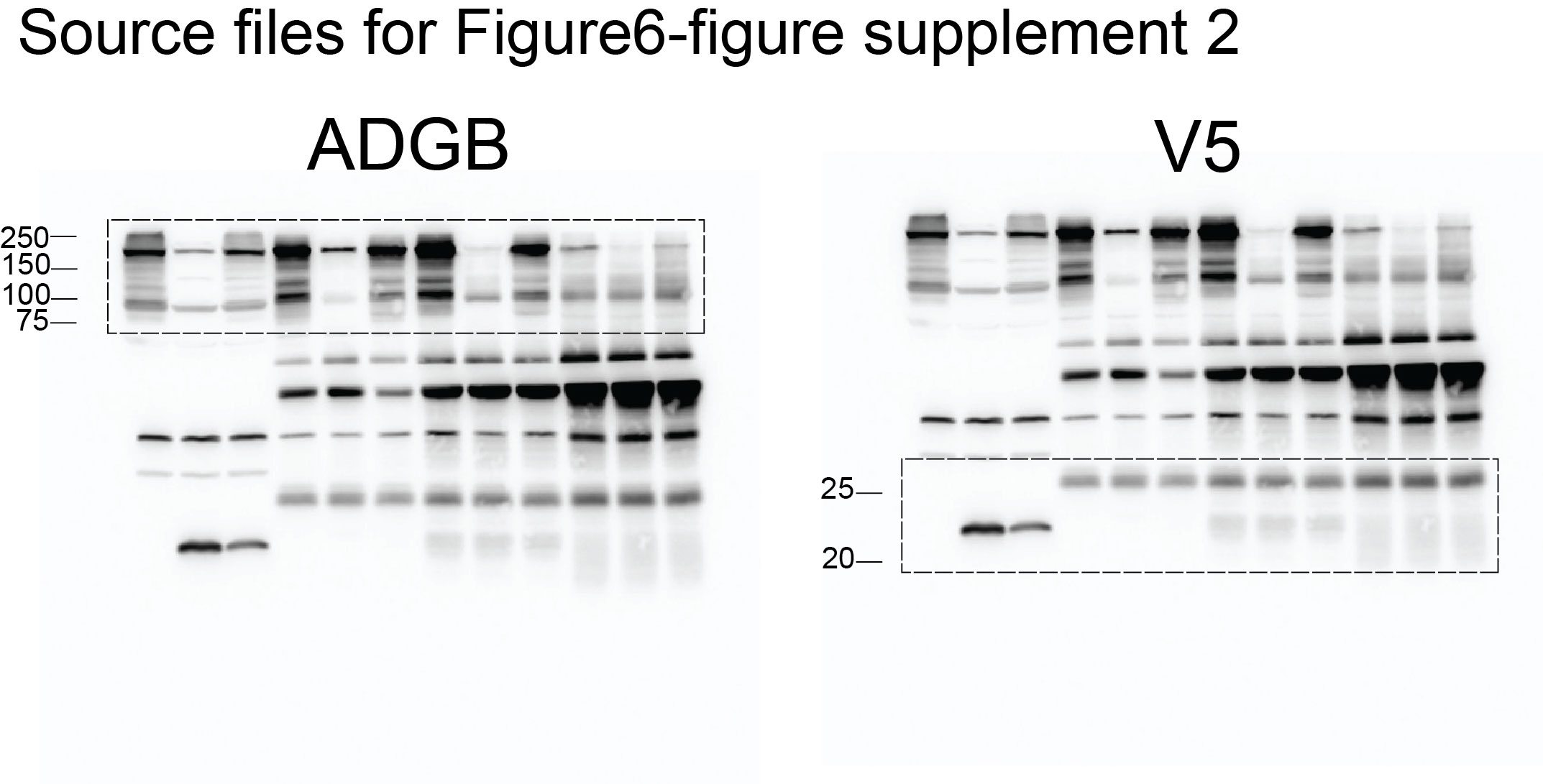

Supplement: Figure 6—figure supplement 2—source data 1. [file elife-72374-fig6-figsupp2-data1.jpg]

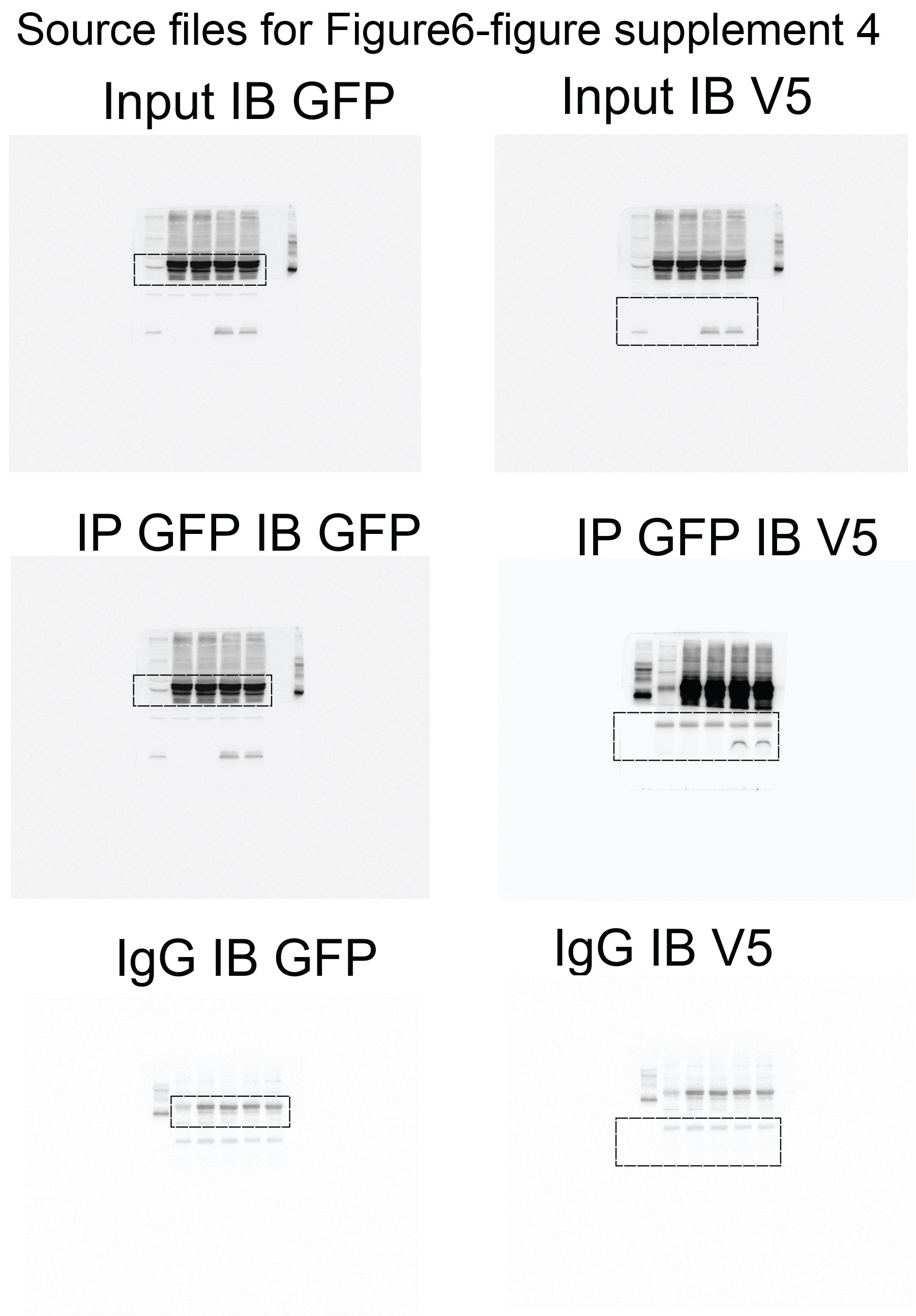

Supplement: Figure 6—figure supplement 4—source data 1. [file elife-72374-fig6-figsupp4-data1.jpg]

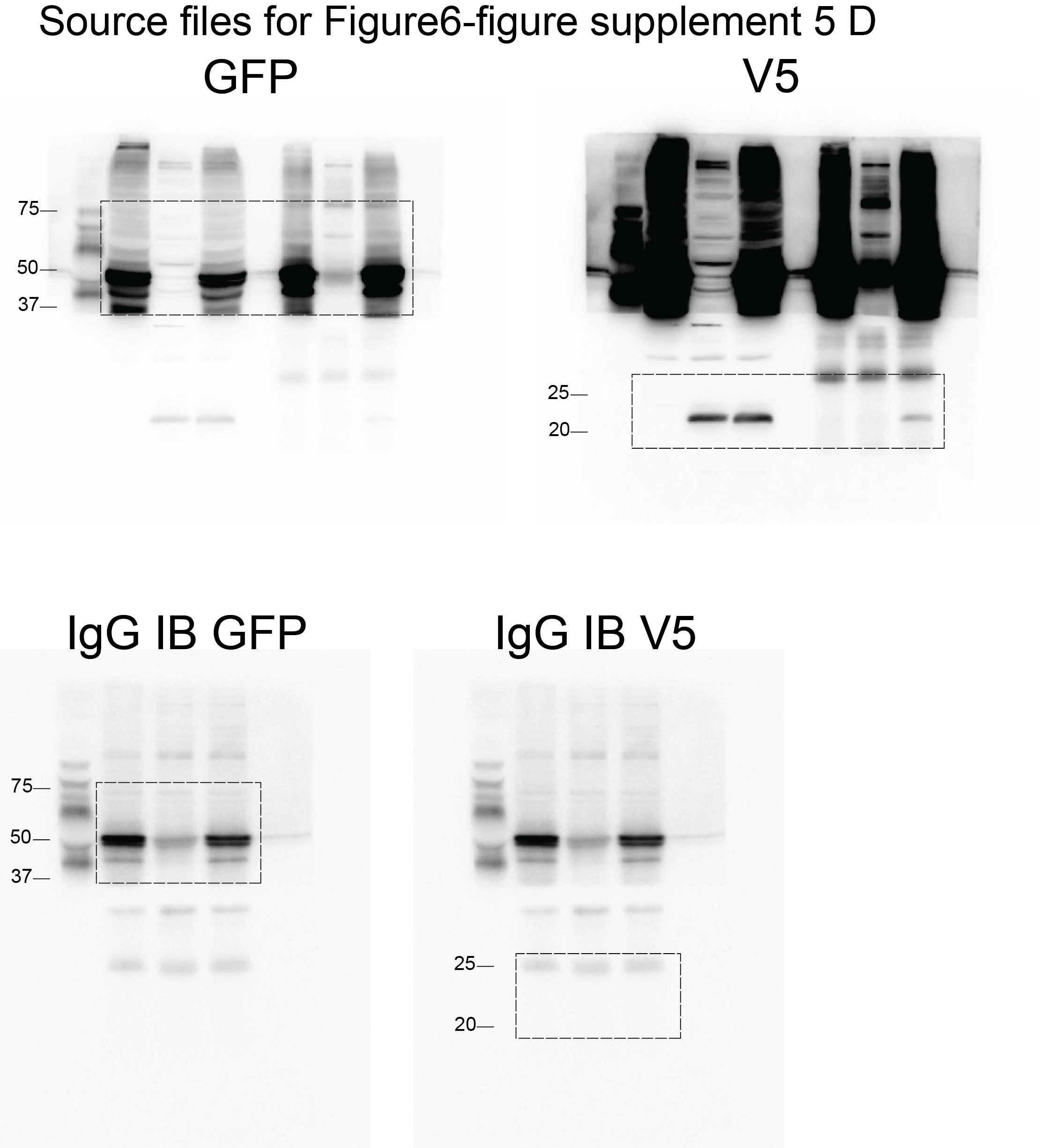

Supplement: Figure 6—figure supplement 5—source data 1. [file elife-72374-fig6-figsupp5-data1.jpg]

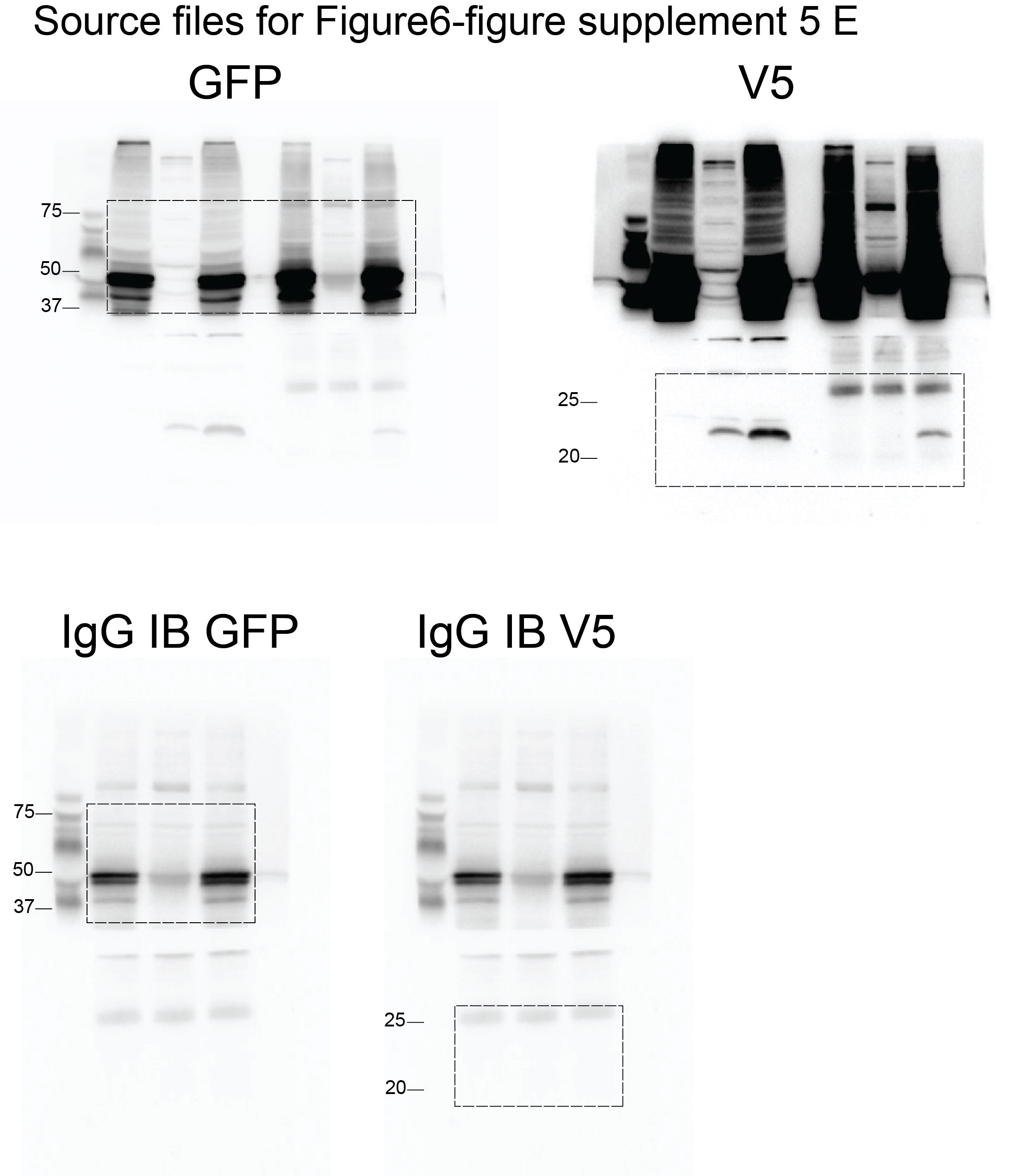

Supplement: Figure 6—figure supplement 5—source data 2. [file elife-72374-fig6-figsupp5-data2.jpg]
