# Supplementary material for: Androglobin, a chimeric mammalian globin, is required for male fertility
Source: eLife. 2022 Jun 14;11:e72374. doi: 10.7554/eLife.72374 (PMC9249397; doi:10.7554/eLife.72374)
Supplement: Supplementary file 1. [file elife-72374-supp1.docx]

List of primers used for RT-qPCR.

| **Gene** | **Forward primer 5’-3’** | **Reverse primer 5’-3’** |
| --- | --- | --- |
| *Adgb* | TTCCAACAGAAACACATTTTGTCCA | TCCATTTACTATGCTCATTTCCCCT |
| *Nos1* | CAGGCAAATCCCAAGCCTATG | CAAAGCACAGCCGAATTTCTC |
| *Nos2* | ATTCACAGCTCATCCGGTACG | GGATCTTGACCATCAGCTTGC |
| *Nos3* | CATTTTCGGACTCACATTGCG | TTGGTCAACCGAACGAAGTG |
| *Sod1* | GTGATTGGGATTGCGCAGTA | TGGTTTGAGGGTAGCAGATGAGT |
| *Sod2* | TTAACGCGCAGATCATGCA | GGTGGCGTTGAGATTGTTCA |
| *Sod3* | CATGCAATCTGCAGGGTACAA | AGAACCAAGCCGGTGATCTG |
| *Bax* | GCGTGGTTGCCCTCTTCTACTTTG | AGTCCAGTGTCCAGCCCATGATG |
| *Bcl2* | AAGGGCTTCACACCCAAATCT | CTTCTACGTCTGCTTGGCTTTGA |
| *Sept10* | GGCCTCATGCGACGAGATAA | CCAATTCCAGTCTCCCCCAC |
| *Actin* | GAGCGTGGCTACAGCTTCAC | GGCATAGAGGTCTTTACGGATG |
| *MMP1* | GCGCACAAATCCCTTCTACC | ATCCGTGTAGCACATTCTGTCC |
| *MMP9* | ATTTCTGCCAGGACCGCTTCTAC | ATCCGGCAAACTGGCTCCTTC |
| *MMP13* | TGCAGAGCGCTACCTGAGATCATAC | GGAGCTTGCTGCATTCTCCTTCA |
| *ACTIN* | CTGGAACGGTGAAGGTGACA | AAGGGACTTCCTGTAACAATGCA |
