# Supplementary material for: Androglobin, a chimeric mammalian globin, is required for male fertility
Source: eLife. 2022 Jun 14;11:e72374. doi: 10.7554/eLife.72374 (PMC9249397; doi:10.7554/eLife.72374)
Supplement: Supplementary file 2. [file elife-72374-supp2.docx]

List of antibodies used throughout the study.

| **Antibody** | **Reference** | **Species** | **Application/dilution** |
| --- | --- | --- | --- |
| anti-mAdgb | Proteintech, custom made against region 409-745 of mAdgb | Rabbit | Immunoblot 1/200  IF 1/300 |
| anti-hADGB N-ter | Atlas Antibodies, HPA036340 | Rabbit | Immunoblot 1/500 |
| anti-hADGB C-ter | OriGene, TA330746 | Rabbit | Immunoblot 1/500 |
| anti-Sept10 | Proteintech, 12420-1-AP | Rabbit | Immunoblot 1/500  IF 1/300 |
| anti-Sept2 | Proteintech, 60075-1-Ig | Mouse | Immunoblot 1/500 |
| anti-Sept7 | Proteintech, 13818-1-AP | Rabbit | Immunoblot 1/500  IF 1/300 |
| anti-Sept8 | Proteintech, 11769-1-AP | Rabbit | Immunoblot 1/500 |
| anti-Sept9 | Proteintech, 10769-1-AP | Rabbit | Immunoblot 1/500 |
| anti-Sept11 | Proteintech, 14672-1-AP | Rabbit | Immunoblot 1/500 |
| anti-Sept14 | Proteintech, 24590-1-AP | Rabbit | Immunoblot 1/500 |
| anti-GFP | Proteintech, 50430-2-AP | Rabbit | Immunoblot 1/1000 |
| anti-V5 | Invitrogen, 46-0705 | Mouse | Immunoblot 1/1000 |
| Anti-FLAG | Sigma F1804 | Mouse | Immunoblot 1/1000 |
| anti-CoxIV | Abcam, ab14744 | Rabbit | IF 1/300 |
| anti-HIF-1α | BD Transduction laboratories, 610958 | Mouse | Immunoblot 1/500 |
| anti-PHD2 | Novus Biologicals, NB100-137 | Rabbit | Immunoblot 1/1000 |
| anti-α−tubulin | Santa Cruz, TU-02 | Mouse | Immunoblot 1/1000 |
